# L-Dopa Comparably Improves Gait and Limb Movements in Parkinson’s Disease: A Wearable Sensor Analysis

**DOI:** 10.3390/biomedicines13112727

**Published:** 2025-11-06

**Authors:** Alessandro Zampogna, Luca Pietrosanti, Giovanni Saggio, Martina Patera, Marco Falletti, Valentina Bellia, Francesco Fattapposta, Giovanni Costantini, Antonio Suppa

**Affiliations:** 1 Department of Human Neurosciences, Sapienza University of Rome, 00185 Rome, Italy; alessandro.zampogna@uniroma1.it (A.Z.); martina.patera@uniroma1.it (M.P.); marco.falletti@uniroma1.it (M.F.); valentina.bellia@uniroma1.it (V.B.); francesco.fattapposta@uniroma1.it (F.F.); 2 IRCCS Neuromed Institute, 86077 Pozzilli, Italy; 3 Department of Electronic Engineering, University of Rome Tor Vergata, 00133 Rome, Italy; ptrlcu02@uniroma2.it (L.P.); saggio@uniroma2.it (G.S.); costantini@uniroma2.it (G.C.)

**Keywords:** Parkinson’s disease, gait, L-Dopa, wearable sensors

## Abstract

**Background/Objectives**: Spatio-temporal gait parameters have been proposed as surrogate markers for objective, remote monitoring of global motor status in Parkinson’s disease (PD). Our observational, cross-sectional pilot study tested whether gait metrics, derived from wearable sensors, reflect dopaminergic responsiveness in both axial and appendicular functions. **Methods**: Twenty-two PD patients were evaluated both under and not under L-Dopa (ON and OFF states, respectively). Motor performance was assessed using wearable inertial sensors during standardized tasks involving gait and upper/lower limb movements. From the recorded kinematics, measures of movement amplitude, speed, rhythm, and consistency were extracted, and dopaminergic response was compared in appendicular and axial functions. **Results**: Treatment effects were more pronounced on the more affected body side. Improvements in appendicular amplitude, speed, and consistency closely matched those observed in spatio-temporal gait parameters. In contrast, rhythm measures displayed a divergent pattern, with reduced gait cadence but increased hand movement frequency, showing an inverse correlation. No significant correlations emerged between axial and appendicular domains for amplitude, velocity, or consistency, whereas improvements in step length and gait velocity were associated with MDS-UPDRS III motor scores. **Conclusions**: These findings overall suggest that specific gait metrics, particularly those reflecting amplitude and velocity, may provide reliable, sensor-based indicators of overall motor status in PD, supporting their use in remote monitoring.

## 1. Introduction

Parkinson’s disease (PD) is a chronic, progressive neurodegenerative disorder characterized by motor signs such as bradykinesia (i.e., reduction in the speed and amplitude of voluntary movements), rigidity (i.e., increased muscle tone during passive joint mobilization), and resting tremor (i.e., involuntary and rhythmic oscillation at rest) [1]. The therapeutic cornerstone of PD remains dopaminergic replacement therapy, based on clinical evaluation and the patient’s subjective report of daily motor fluctuations [1]. In early disease stages, this approach is generally sufficient, as motor performance observed during in-clinic visits tends to reflect the patient’s real-life condition. However, with disease progression, clinical management becomes increasingly challenging due to the emergence of motor fluctuations (i.e., alternating ON states with improved mobility and OFF states with re-emergence of motor symptoms) and L-Dopa induced dyskinesias (i.e., drug-related involuntary movements) [2]. These fluctuations are closely tied to medication timing and are often variable or unpredictable, complicating treatment adjustments and disease control [3]. As a result, the therapeutic strategy in moderate-to-advanced PD commonly relies heavily on patient-reported diaries, which attempt to capture symptom variability in daily life [4]. While diaries offer the advantage of contextual insight, they are inherently subjective, prone to recall bias, and limited in their ability to quantify symptom severity or treatment response [5,6].

Over the last three decades, wearable technologies have emerged as promising tools to objectively monitor motor issues in PD [7,8,9,10,11]. Among these, inertial measurement units (IMUs) allow for accurate reconstruction of body movements through real-time motion sensing. These devices are usually favored for their portability, ease of setup, and suitability for free-living applications outside laboratory settings [12]. Several IMU-based systems have received regulatory approval and are being integrated into clinical workflows [13]. Their use enables continuous, ecologically valid monitoring in real-world settings, capturing motor function during daily activities, which provides a more comprehensive picture than the time-limited hospital assessment [14,15,16]. Traditional assessment systems typically employ multiple sensors (commonly ranging from 2 to 17 devices) placed on different body segments [12]. To maximize usability and facilitate adoption of these technologies in unsupervised settings by individuals without technical expertise, it is essential to minimize the number of required sensors while preserving the sensitivity and clinical relevance of the data collected. Among proposed strategies, gait monitoring via one or two IMUs has gained traction, based on the hypothesis that gait dynamics can serve as a reliable marker of overall clinical status [17,18,19,20]. Previous studies have shown that spatio-temporal gait parameters, such as velocity, stride length and duration, are responsive to dopaminergic therapy and vary according to motor states [21,22]. Furthermore, these parameters have been linked to disease severity and progression, reinforcing their potential as surrogate markers of motor function in PD [23,24,25]. However, gait represents a predominantly axial function, and it remains unclear whether its dopaminergic responsiveness mirrors that of appendicular (limb) motor tasks. Standard clinical assessments such as the MDS-UPDRS distinguish between axial and appendicular motor domains (e.g., gait and postural instability vs. tremor, rigidity, and limb bradykinesia). This distinction is clinically relevant, as axial and appendicular symptoms may follow different pathophysiological mechanisms and therapeutic responses. To date, no study has systematically examined whether axial and appendicular responses to dopaminergic therapy have overlapping trajectories, or whether gait metrics alone can capture the broader spectrum of motor impairment in PD. Exploring the relationship between gait parameters and appendicular motor measures may therefore clarify whether gait metrics can serve as reliable clinical biomarkers of overall motor status and provide a conceptual basis for reducing the number of sensors required for long-term remote monitoring in PD.

In this observational, cross-sectional pilot study, we used wearable IMUs to objectively assess whether gait-related parameters reliably capture the motor response across different body segments in PD, focusing on movement amplitude, speed, rhythm, and consistency. Accordingly, the primary objective was to compare the percentage improvement in axial (gait and postural sway) and appendicular (e.g., hand and leg movements) functions following dopaminergic therapy. As a secondary objective, we examined whether the percentage improvements were correlated, thereby exploring the extent to which gait responsiveness reflects the broader motor benefit. We hypothesize that gait metrics may serve as a robust and comprehensive indicator of overall motor status in PD, with potential relevance for remote and home-based monitoring.

## 2. Materials and Methods

This observational, cross-sectional, pilot study was conducted following the guidelines outlined in the “Strengthening the Reporting of Observational Studies in Epidemiology” (STROBE) statement, ensuring adherence to standardized methodological and reporting criteria (Supplementary Materials S1) [26].

### 2.1. Subjects and Clinical Evaluation

A cohort of 22 individuals with PD was prospectively enrolled at the Movement Disorders outpatient clinic of Sapienza University of Rome, Italy. Inclusion criteria comprised a diagnosis of idiopathic PD based on established international guidelines, Hoehn and Yahr stage ≤3 [27], preserved independent ambulation and upright posture, absence of dementia (as indicated by a MoCA score >23) [28], and no comorbidities interfering with gait or upper limb motor function. Patients with atypical parkinsonism were excluded from the study. Also, those with freezing of gait, defined as a brief, episodic inability or marked reduction in forward progression of the feet despite the intention to walk, were excluded to ensure a more homogeneous sample, given the substantial impact of this phenomenon on gait [29]. Comprehensive clinical evaluation was conducted using a standardized battery, including the Hoehn and Yahr scale, the Movement Disorder Society—Unified Parkinson’s Disease Rating Scale part III (MDS-UPDRS III), the Montreal Cognitive Assessment (MoCA), and the Frontal Assessment Battery (FAB) [30]. Each participant was assessed in two motor conditions: the OFF state, after overnight withdrawal from dopaminergic medications (at least 12 h), and the ON state, approximately one hour after administration of 150% of their usual morning dose of L-Dopa, consistent with prior studies employing supratherapeutic challenges to characterize dopaminergic responsiveness [31,32,33]. The L-Dopa equivalent daily dose (LEDD) was calculated according to established conversion methods [34]. None of the patients were receiving medications other than dopaminergic therapy, including any neuropsychiatric drugs, at the time of the study. Moreover, all patients were on stable dopaminergic treatment for at least four weeks prior to participation. Demographic and clinical characteristics of the cohort are reported in Table 1. All participants provided written informed consent before inclusion, and the study received approval from the local ethics committee of Sapienza University of Rome in accordance with the Declaration of Helsinki (protocol code 0372/2022; date of approval: 10 May 2022).

### 2.2. Experimental Procedures

Following the clinical assessment, all patients underwent a standardized instrumental evaluation of motor performance based on specific motor tasks derived from selected items of the MDS-UPDRS part III, targeting both appendicular and axial motor function.

**Figure 1:**
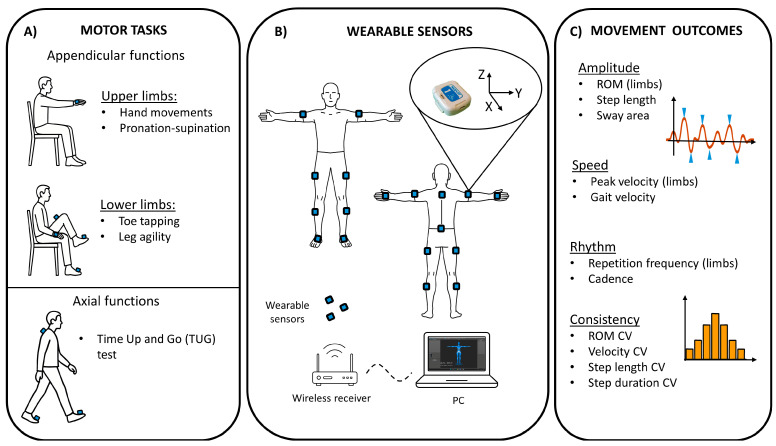
Experimental design. (**A**) Summary of the standardized motor tasks performed to assess appendicular and axial motor functions. (**B**) Placement of wearable inertial sensors on the participants’ body segments and schematic representation of the data acquisition process, with wireless transmission from the sensors to a computer via a receiver. (**C**) Summary of the kinematic outcome measures considered for appendicular and axial motor functions.

To assess appendicular motor abilities, the following tasks were performed (ten times at maximal speed and amplitude, as specified in the corresponding MDS-UPDRS items): (1) hand movements (rapidly tapping the hand while keeping the wrist in contact with a flat surface, resembling item 3.5 of the MDS-UPDRS, namely hand opening and closing); (2) pronation-supination movements of hands (item 3.6, requiring repetitive turning the palm up and down alternately); (3) toe tapping (item 3.7, which involves rapidly tapping the toes while the heel remains in contact with the ground); (4) leg agility (item 3.8, consisting of repetitive up-and-down tapping of the foot while keeping the heel lifted) (Figure 1A).

For axial motor evaluation, patients completed a linear walking task modelled on the Timed Up and Go test, which integrates MDS-UPDRS items 3.9 and 3.10. This composite task required participants to rise from a chair, walk a 6 m straight path, turn 180 degrees, return, and sit down. In addition, postural sway during walking was assessed by analyzing medio-lateral and antero-posterior oscillations of the trunk, as a proxy of dynamic postural control. All motor tasks were performed both during the OFF and ON medication states (Figure 1A).

### 2.3. Wearable Sensors and Instrumental Outcome Measures

For the standardized instrumental assessment of motor performance, thirteen inertial sensors termed Movit G1 (by Captiks Srl, Rome, Italy; ≈20 g, 4 × 3 × 1.5 cm) were secured with Velcro™ straps to the posterior trunk, bilateral upper arms, forearms, thighs, shanks, and the dorsal surfaces of the hands and feet. Each unit’s validated IMU (triaxial accelerometer ±8 g, 16 384 LSB/g; triaxial gyroscope ±2000°/s, 32.8 LSB/°/s) streamed acceleration, angular velocity, and orientation data at a sample rate of 52 Hz to a laptop receiver [35]. Raw signals were first processed with Motion Studio software G1 environment (Captiks Srl) to derive joint angles. Subsequently, the data were analyzed in MATLAB R2025b (The MathWorks Inc., Natick, MA, USA), where a moving average filter with a 10-sample window was applied to smooth the signals. Prior to each recording session, wearable sensors were calibrated following the manufacturer’s recommended procedures [36]. These included static alignment, execution of defined motion sequences for sensor-to-segment alignment, and synchronization across all sensor channels, overall ensuring accuracy of segment orientation estimates and inter-sensor timing alignment (Figure 1B).

Concerning instrumental outcome measures, task-specific kinematic and spatio-temporal metrics were considered. For upper- and lower-limb maneuvers (appendicular functions), range of motion (ROM, i.e., the total angular excursion of the joint during the movement cycle), peak velocity (i.e., the highest instantaneous speed reached during a movement), repetition frequency (i.e., the number of complete movement cycles performed per second) and coefficients of variation (CV) for both ROM and velocity were extracted to characterize movement amplitude, speed, and consistency. ROM and velocity values were averaged across trials and analyzed separately for the more and less affected sides (defined according to the relative motor scores of each hemibody in the MDS-UPDRS part III). Gait performance was assessed by measuring spatio-temporal parameters, including cadence (i.e., number of steps per minute), step velocity (i.e., distance covered per unit of time during walking), duration (i.e., the time elapsed between the initial contact of one foot and the initial contact of the opposite foot), and length (i.e., the distance between successive heel strikes of opposite feet), as well as their cycle-to-cycle variability (CV of step duration and length). Dynamic postural control during ambulation was indexed by the sway area (i.e., the area encompassed by medio-lateral and antero-posterior trunk oscillations during gait), in line with established protocols [9] (Figure 1C).

### 2.4. Statistical Analysis

Since data distribution did not satisfy the assumptions required for parametric analyses, as assessed by the Shapiro–Wilk normality test, non-parametric statistical methods were employed [37]. Descriptive statistics were used to summarize the clinical and demographic characteristics of the patient cohort. For each instrumental outcome measure, the therapeutic response to dopaminergic medication was computed as the percentage change according to the following formula:

$$var\%\;=\;\left[\frac{ON\;-\;OFF}{OFF}\right]\times \;100$$

where ON indicates measurements recorded one hour after L-Dopa intake, whereas OFF refers to the unmedicated condition. This index expresses the magnitude of improvement induced by dopaminergic treatment relative to the baseline OFF state.

For appendicular tasks, outcome measures were compared between the more and the less affected sides (side exhibiting greater and lower baseline motor impairment, respectively) using the Wilcoxon signed-rank test. Additionally, to explore potential differences in therapeutic responsiveness based on body topography (appendicular vs. axial functions), the Friedman test was performed, followed by Wilcoxon signed-rank tests for pairwise comparisons when appropriate [38]. To allow a meaningful comparison between appendicular and axial domains, dopaminergic responsiveness was examined across homologous kinematic features: ROM of upper and lower limbs tasks was compared with step length and sway area (movement amplitude), peak velocity with step velocity (movement speed), repetition frequency with cadence (movement rhythm), and the CV of ROM and velocity with the CV of step length and duration (movement consistency).

Spearman’s rank correlation coefficient was finally used to investigate associations between percentage changes in kinematic parameters as well as with corresponding clinical score variations [39]. To control the false discovery rate and limit the likelihood of Type I errors arising from multiple comparisons, the Benjamini–Hochberg correction was applied to the entire set of statistical tests within each analysis, and only adjusted *p*-values are reported as significant [40]. No missing data were present in the dataset.

Statistical analyses were conducted using IBM SPSS Statistics, Version 29.0 (IBM Corp., Armonk, NY, USA).

## 3. Results

### 3.1. Clinical Features

Clinical and demographic characteristics of the enrolled PD patients are summarized in Table 1. All participants successfully completed experimental procedures without complications or adverse events. Testing was performed under uniform environmental conditions, and all participants demonstrated good compliance with task instructions. No participants withdrew from the study, and data collection was feasible across all assessment sessions, both OFF and ON state of therapy.

### 3.2. Sensor-Derived Measures

When comparing the dopaminergic response of appendicular tasks between the more and less affected sides using Wilcoxon signed-rank tests, overall changes were comparable between sides, except for specific parameters. Specifically, greater improvements were observed on the more affected side for movement frequency during hand opening/closing (Z = −1.997, *p* = 0.046), ROM during pronation–supination (Z = −2.127, *p* = 0.033), and peak velocity during leg agility (Z = −2.159, *p* = 0.031), compared with the less affected side. No other significant side-to-side differences were observed for the remaining kinematic parameters or tasks, including foot tapping (all *p* > 0.05). Given that the largest treatment-induced changes were consistently observed on the more affected side, subsequent analyses and correlations with axial measures were conducted using data from this side only.

**Figure 2:**
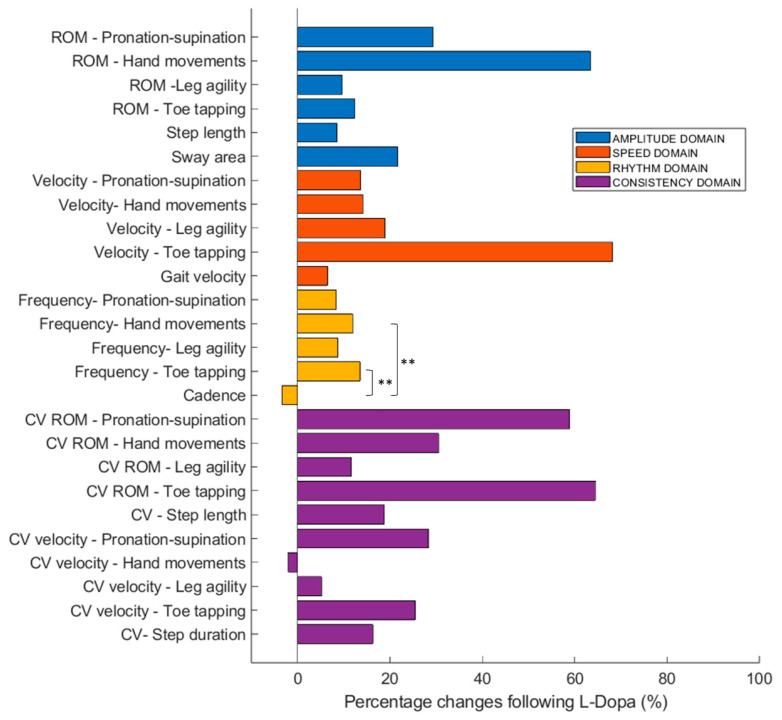
Kinematic Changes After L-Dopa administration. Bar plots illustrate the percentage change in kinematic outcome measures following L-Dopa administration for appendicular and axial functions. Measures are grouped by functional domains, including movement amplitude, speed, rhythm, and consistency. Asterisks indicate statistically significant differences (** *p* < 0.01).

In line with the study hypothesis that gait-related measures may reflect overall motor performance in PD, the Friedman analyses showed no significant differences in the percentage change after dopaminergic therapy for movement amplitude (ROM of hand movements, hand pronation–supination, toe tapping, leg agility, step length, and sway area; χ^2^(5) = 10.088, *p* = 0.073) or for movement speed (velocity of hand movements, hand pronation–supination, toe tapping, leg agility, and gait speed; χ^2^(4) = 4.610, *p* = 0.330). In contrast, a significant effect was found for movement rhythm, including the frequency of hand movements, hand pronation–supination, toe tapping, leg agility, and gait cadence (χ^2^(4) = 9.670, *p* = 0.046). No significant differences were detected for movement consistency, assessed through the CV of ROM and velocity for upper- and lower-limb tasks as well as for step length, duration, and velocity (χ^2^(9) = 2.833, *p* = 0.971). Post hoc Wilcoxon tests on rhythm measures revealed a divergent pattern, with a slight reduction in gait cadence contrasting with increased frequencies of hand movements (Z = −2.972, *p* = 0.003) and toe tapping (Z = −3.146, *p* = 0.002), while no significant differences were observed for hand pronation–supination (Z = −1.095, *p* = 0.274) or leg agility (Z = −1.616, *p* = 0.106). These differences remained significant after Benjamini–Hochberg correction (adjusted *p* = 0.006 for both hand movements and toe tapping). Table 2 reports the mean percentage improvements in the evaluated measures, which are also illustrated in Figure 2 by movement domain (amplitude, velocity, rhythm, and consistency).

### 3.3. Clinical-Behavioral Correlations

Spearman’s correlation analysis showed no significant associations between appendicular and axial parameters for movement amplitude, velocity, or consistency after Benjamini–Hochberg correction (all *p* > 0.05). For movement rhythm, a significant inverse association emerged between hand movement frequency and gait cadence (ρ = −0.43, adjusted *p* = 0.01). Finally, concerning correlations with clinical scores, step length and gait velocity both showed positive associations with MDS-UPDRS III scores in the OFF state (R = 0.50 for both, adjusted *p* = 0.022), whereas in the ON state only step length remained significantly correlated (R = 0.49, adjusted *p* = 0.045).

## 4. Discussion

In this observational, cross-sectional pilot study, by using a network of wearable inertial sensors, we quantitatively demonstrated that, although not directly correlated, the percentage change in various functional domains of appendicular (limb) movements is comparable to that observed in gait in patients with PD. Moreover, kinematic parameters of gait show significant associations with motor changes clinically evaluated through standardized scales in PD.

To minimize potential confounding factors and ensure the reliability of our findings, we adopted strict clinical inclusion criteria and rigorous methodological procedures. Patients with comorbidities potentially influencing motor performance, such as orthopedic, rheumatologic, or cognitive impairment, were excluded to isolate disease-related motor features. All assessments were conducted in a well-defined OFF- and ON-medication states, in accordance with established protocols usually adopted in clinical trials [41]. Moreover, we employed validated wearable inertial sensors previously benchmarked against gold standard motion capture systems [42]. To ensure consistency and measurement accuracy, sensors were individually calibrated for each participant prior to every recording session.

As a first finding, the degree of improvement in spatio-temporal parameters related to appendicular function was largely comparable between the more and less affected sides, except for specific amplitude and velocity measures, which showed greater improvement on the more affected side. This result is not unexpected, as it is plausible that a higher baseline level of motor impairment in the more affected limbs allowed for greater observable changes in response to dopaminergic therapy [43]. This finding aligns with a well-known rehabilitation observation whereby more impaired functions have greater potential for measurable improvement, often referred to as the ‘greater room for improvement’ principle [44]. Also, it reflects the asymmetric nature of PD, with dopaminergic therapy exerting greater relative effects on the more impaired side [45]. In line with this concept, we observed that higher MDS-UPDRS III scores in the OFF state were associated with greater improvements in step length and gait velocity. This rationale overall supports our decision to focus subsequent analyses on the more affected side, where treatment-related changes are more likely to emerge, fully in line with previous experimental evidence [46,47,48].

### 4.1. L-Dopa Comparably Improves Gait and Limb Movements in PD

When looking at kinematic changes following dopaminergic therapy at appendicular and axial body segments, movement amplitude, velocity, and consistency improved to a comparable extent across limb tasks and gait. This result suggests that dopaminergic therapy exerts a relatively uniform effect across both appendicular and axial motor functions in the mid-stage of the disease, despite distinct anatomical substrates and patterns of degeneration [49,50]. Our findings are fully consistent with previous evidence showing that dopaminergic therapy can significantly enhance key spatio-temporal gait parameters, including step length, gait speed, and stride stability, as well as improve upper limb movement amplitude and fine motor control during tasks such as hand tapping and reach-to-grasp movements [21,51,52,53,54]. From a neurophysiological perspective, axial muscles are predominantly controlled by brainstem descending pathways (e.g., vestibulospinal, reticulospinal, and interstitio-spinal tracts) while appendicular muscles are primarily governed by the more recently evolved lateral motor system, including corticospinal and rubrospinal tracts [55]. The comparable responsiveness to L-Dopa of appendicular functions and gait may therefore reflect partially shared functional networks and common dopaminergic modulation of both systems by basal ganglia circuits [54]. Despite similar average improvements, we unexpectedly found no significant associations between amplitude, velocity, and consistency changes in appendicular movements and gait. The lack of significant correlations between individual improvements suggests that these functions may respond differently on a patient-by-patient basis, with a general similarity but not always a one-to-one correspondence. This may appear contradictory to the idea that gait is a comprehensive indicator of overall motor status, yet, it likely reflects individual variability in response to L-Dopa, heterogeneity in disease progression patterns, or differences in measurement accuracy. In addition, asymmetry in symptom distribution and variations in the underlying neurodegenerative burden across motor domains may have further contributed to the lack of correlations. Nevertheless, the comparable group-level improvement across tasks still supports a shared dopaminergic influence on both domains and suggests that gait metrics may sensitively capture clinically relevant motor phenomena, such as fluctuations, thus remaining informative of the overall motor performance.

In contrast to movement amplitude, velocity, and consistency, movement rhythm exhibited a task-specific response pattern. While the frequency of repetitive upper- and lower-limb movements increased following dopaminergic therapy, gait cadence decreased slightly, suggesting an opposite clinical response in this motor domain. Previous studies have similarly reported an increase in the frequency of repetitive upper- and lower-limb movements, accompanied by a reduction in gait cadence in the ON state compared with the OFF state of dopaminergic therapy [54,56,57,58,59]. This discrepancy was also confirmed by an inverse correlation between these measures in our cohort of patients and likely reflects the recruitment of different motor strategies depending on the specific motor task. Indeed, in unconstrained limb movements, the reduction in bradykinesia translates directly into a higher execution rate [60]. By contrast, during walking, the same dopaminergic effect appears to promote longer and more efficient steps, resulting in a modest decrease in cadence rather than an acceleration of the stepping rhythm [61]. An alternative hypothesis is that L-Dopa exerts a more pronounced effect on the spatial features of gait, whereas temporal parameters such as cadence remain comparatively less responsive [62]. These observations are fully in line with current neurophysiological evidence indicating that, while basal ganglia play a predominant role in regulating spatial aspects of movement, such as amplitude and velocity, temporal features like rhythm rely more heavily on non-dopaminergic networks, including brainstem and cerebellar circuits [63,64]. Indeed, neuronal activity in key basal ganglia output nuclei, including the *globus pallidus pars interna* and the *subthalamic nucleus*, has been shown to scale with movement vigor, encompassing both force and speed, and to adjust motor output according to motivational and contextual cues [65,66]. Also, dopaminergic therapy and interventions targeting the basal ganglia (e.g., deep brain stimulation) in PD typically produce marked improvements in step length, gait speed, and overall movement amplitude, while rhythm-related parameters, such as cadence, often remain less responsive [62,67]. Overall, the divergent pattern of rhythm measures in appendicular movements and gait suggests that these may not represent a reliable clinical biomarker for monitoring the overall motor status in PD through wearable sensors.

### 4.2. Gait as a Clinical Biomarker of Disease State

The observation of comparable changes in movement amplitude, velocity, and consistency across limb functions and gait parameters supports the use of gait analysis as a robust and comprehensive indicator of overall motor status in PD [68,69,70]. To date, several wearable sensor systems have already been proposed for long-term gait monitoring in PD with potential applications in home-based settings [13,71,72]. Our findings further strengthen the validity of this approach, which may enable the automated and quantitative collection of ecological data that dynamically capture the patient’s motor condition during daily life activities [73]. This, in turn, could facilitate the optimization and personalization of therapeutic strategies. It should be noted, however, that in this study, appendicular and axial measures were assessed under supervised conditions using a wearable sensor network composed of multiple devices. This was methodologically necessary to ensure the most accurate and reliable quantification of each motor domain, in line with the study objectives. It remains to be established whether a smaller set of sensors, as typically used in free-living settings, can achieve similar accuracy in gait metric estimation. Moreover, in our cohort we observed a marked interindividual variability in the percentage change in several metrics, likely reflecting the heterogeneous clinical expression and treatment responsiveness of PD symptoms. This variability may represent a significant challenge for clinical applications and emphasizes the need for individualized baselines and longitudinal within-patient monitoring to improve the interpretability and reliability of sensor-derived measures in PD. Nevertheless, the significant correlation observed between changes in spatio-temporal gait parameters and severity of motor impairment clinically assessed through standardized scales (MDS-UPDRS III) further reinforces the validity of using specific gait metrics, particularly those related to amplitude and velocity, as reliable biomarkers of overall motor status in patients with PD. Taken together, these findings encourage future studies aimed at validating minimal wearable sensor setups for real-world monitoring, with the goal of integrating kinematic biomarkers into routine clinical practice.

When evaluating the present study, some limitations should be acknowledged. First, this study was an exploratory analysis and the relatively small sample size (*n* = 22) may have limited the statistical power of our analyses, warranting confirmation in larger cohorts. Second, our cohort consisted of patients in the mid-stage of PD, which may limit the generalizability of the results. Indeed, in more advanced disease stages, the response to dopaminergic therapy may differ across axial and appendicular domains due to the emergence of L-Dopa–resistant symptoms, such as postural instability and freezing of gait. Third, none of the patients here examined exhibited freezing of gait (i.e., a paroxysmal disturbance that can directly affect spatio-temporal gait parameters). Given that this phenomenon and its responsiveness to L-Dopa may significantly influence gait outcomes, its absence in our cohort might have contributed to a more homogeneous response to treatment and to the overall stability of gait parameters observed. Accordingly, future studies should specifically account for its presence and potential impact, especially in patients with advanced disease.

## 5. Conclusions

In this cross-sectional, observational pilot study, specific measures of movement amplitude, velocity, and consistency, but not rhythm, showed a comparable response to dopaminergic therapy across both appendicular motor functions and gait. Within these domains, gait parameters such as step length, velocity, and their CV emerged as promising kinematic biomarkers for monitoring the clinical status of patients with PD. Overall, these findings support the use of wearable-derived gait metrics as objective markers of dopaminergic response across different body regions, complementing traditional clinical assessments in PD. These metrics can, therefore, be incorporated into routine clinical and research assessments to enable objective and continuous monitoring of motor fluctuations and treatment responsiveness in real-world conditions. Such integration may also pave the way for practical applications, including electronically compiled motor diaries based on gait monitoring, potentially enhanced by machine learning algorithms to detect clinically relevant fluctuations. Research should now aim to validate these biomarkers in larger, more heterogeneous cohorts, extend assessments to real-life, home-based environments, and determine their predictive value for disease progression and treatment optimization. Longitudinal studies are also required to establish whether the dopaminergic responsiveness of spatial gait features is preserved or altered over the course of the disease, and to explore their ability to predict key clinical milestones such as the onset of freezing of gait, increased fall risk, or the need for advanced therapies.

## Figures and Tables

**Table 1:** Demographic and clinical characteristics of patients with Parkinson’s disease (mean and standard deviation).

Participants (N)	Age	Disease Duration	Hoehn and Yahr	MDS-UPDRS III		Clinical Phenotype	MoCA	FAB	LEDD
				OFF	ON				
14 Males 8 Females	70.4 ± 7.9	3.9 ± 3.5	2.1 ± 0.5	30.8 ± 9.7	19.4 ± 10.1	13 AR 6 Trem 3 Mixed	25.5 ± 3.4	15.4 ± 2.5	513.2 ± 226.8

AR: akinetic/rigid phenotype; FAB: frontal assessment battery; LEDD: L-Dopa equivalent daily dose; MoCA: Montreal cognitive assessment; Trem: tremor-dominant phenotype.

**Table 2:** Percentage change in sensor-based measures following L-Dopa intake in patients with Parkinson’s disease.

	Motor Tasks	Movement Amplitude	Movement Speed	Movement Rhythm	Movement Consistency
**Appendicular functions** **(more affected side)**	Hand movements	ROM: 63.4 ± 154.9	Vel: 14.1 ± 34.6	Freq: 11.9 ± 17.1	CV ROM: 30.5 ± 136.7 CV vel: −2.0 ± 43.7
	Hand pronation–supination	ROM: 29.3 ± 83.8	Vel: 13.6 ± 64.1	Freq: 8.4 ± 35.7	CV ROM: 58.9 ± 234.7 CV vel: 28.3 ± 74.9
	Toe tapping	ROM: 12.4 ± 65.4	Vel: 68.2 ± 324.4	Freq: 13.5 ± 21.1	CV ROM: 64.4 ± 245.6 CV vel: 25.5 ± 89.0
	Leg agility	ROM: 9.7 ± 102.8	Vel: 18.9 ± 63.2	Freq: 8.7 ± 26.7	CV ROM: 11.7 ± 64.9 CV vel: 5.2 ± 31.9
**Axial functions**	Gait	Step length: 3.9 ± 11.1	Step vel: 6.5 ± 16.5	Cad: −3.4 ± 5.4	CV step length: 18.7 ± 104.6

Cad: cadence; CV: coefficient of variation; Freq: frequency; ROM: range of motion; Vel: velocity.

## Data Availability

The original contributions presented in this study are included in the article/supplementary material. Further inquiries can be directed to the corresponding author.

## Supplementary Materials

The following supporting information can be downloaded at: https://www.mdpi.com/article/10.3390/biomedicines13112727/s1, Supplementary Material S1: STROBE checklist.

## Abbreviations

The following abbreviations are used in this manuscript:

CV	Coefficient of Variation
FAB	Frontal Assessment Battery
IMU	Inertial Measurement Unit
LEDD	L-Dopa Equivalent Daily Dose
MDS-UPDRS	Movement Disorder Society—Unified Parkinson’s Disease Rating Scale
MoCA	Montreal Cognitive Assessment
PD	Parkinson’s Disease
ROM	Range of Motion
STROBE	Strengthening the Reporting of Observational Studies in Epidemiology

## References

[1] Tanner C.M., Ostrem J.L. (2024). Parkinson’s Disease. N. Engl. J. Med..

[2] Aradi S.D., Hauser R.A. (2020). Medical Management and Prevention of Motor Complications in Parkinson’s Disease. Neurotherapeutics.

[3] Regensburger M., Csoti I., Jost W.H., Kohl Z., Lorenzl S., Pedrosa D.J., Lingor P. (2025). Motor and Non-Motor Fluctuations in Parkinson’s Disease: The Knowns and Unknowns of Current Therapeutic Approaches. J. Neural Transm..

[4] Antonini A., Martinez-Martin P., Chaudhuri R.K., Merello M., Hauser R., Katzenschlager R., Odin P., Stacy M., Stocchi F., Poewe W. (2011). Wearing-off Scales in Parkinson’s Disease: Critique and Recommendations. Mov. Disord..

[5] Amanzio M., Monteverdi S., Giordano A., Soliveri P., Filippi P., Geminiani G. (2010). Impaired Awareness of Movement Disorders in Parkinson’s Disease. Brain Cogn..

[6] Papapetropoulos S.S. (2012). Patient Diaries as a Clinical Endpoint in Parkinson’s Disease Clinical Trials. CNS Neurosci. Ther..

[7] Moreau C., Rouaud T., Grabli D., Benatru I., Remy P., Marques A.-R., Drapier S., Mariani L.-L., Roze E., Devos D. (2023). Overview on Wearable Sensors for the Management of Parkinson’s Disease. npj Park. Dis..

[8] Zampogna A., Manoni A., Asci F., Liguori C., Irrera F., Suppa A. (2020). Shedding Light on Nocturnal Movements in Parkinson’s Disease: Evidence from Wearable Technologies. Sensors.

[9] Zampogna A., Mileti I., Palermo E., Celletti C., Paoloni M., Manoni A., Mazzetta I., Dalla Costa G., Pérez-López C., Camerota F. (2020). Fifteen Years of Wireless Sensors for Balance Assessment in Neurological Disorders. Sensors.

[10] Ricci M., Lazzaro G.D., Errico V., Pisani A., Giannini F., Saggio G. (2022). The Impact of Wearable Electronics in Assessing the Effectiveness of Levodopa Treatment in Parkinson’s Disease. IEEE J. Biomed. Health Inform..

[11] Lobo P., Morais P., Murray P., Vilaça J.L. (2024). Trends and Innovations in Wearable Technology for Motor Rehabilitation, Prediction, and Monitoring: A Comprehensive Review. Sensors.

[12] Rast F.M., Labruyère R. (2020). Systematic Review on the Application of Wearable Inertial Sensors to Quantify Everyday Life Motor Activity in People with Mobility Impairments. J. Neuroeng. Rehabil..

[13] Rodríguez-Martín D., Pérez-López C. (2024). Commercial Symptom Monitoring Devices in Parkinson’s Disease: Benefits, Limitations, and Trends. Front. Neurol..

[14] Zampogna A., Borzì L., Rinaldi D., Artusi C.A., Imbalzano G., Patera M., Lopiano L., Pontieri F., Olmo G., Suppa A. (2024). Unveiling the Unpredictable in Parkinson’s Disease: Sensor-Based Monitoring of Dyskinesias and Freezing of Gait in Daily Life. Bioengineering.

[15] Zampogna A., Borzì L., Soares C., Demrozi F. (2024). Editorial: High-Tech Personalized Healthcare in Movement Disorders. Front. Neurol..

[16] Moon S.H., Soangra R., Frames C.W., Lockhart T.E. (2021). Three Days Monitoring of Activities of Daily Living among Young Healthy Adults and Parkinson’s Disease Patients. Biomed. Sci. Instrum..

[17] Mancini M., Afshari M., Almeida Q., Amundsen-Huffmaster S., Balfany K., Camicioli R., Christiansen C., Dale M.L., Dibble L.E., Earhart G.M. (2025). Digital Gait Biomarkers in Parkinson’s Disease: Susceptibility/Risk, Progression, Response to Exercise, and Prognosis. npj Park. Dis..

[18] Di Filippo F., De Biasi G., Russo M., Ricciardi C., Pisani N., Volzone A., Aiello M., Cuoco S., Calabrese M., Romano M. (2025). Dual-Task-Related Gait Patterns as Possible Marker of Precocious and Subclinical Cognitive Alterations in Parkinson Disease. Sci. Rep..

[19] Del Din S., Godfrey A., Mazzà C., Lord S., Rochester L. (2016). Free-Living Monitoring of Parkinson’s Disease: Lessons from the Field. Mov. Disord..

[20] Raschka T., To J., Hähnel T., Sapienza S., Ibrahim A., Glaab E., Gaßner H., Steidl R., Winkler J., Corvol J.-C. (2025). Objective Monitoring of Motor Symptom Severity and Their Progression in Parkinson’s Disease Using a Digital Gait Device. Sci. Rep..

[21] Suppa A., Kita A., Leodori G., Zampogna A., Nicolini E., Lorenzi P., Rao R., Irrera F. (2017). L-DOPA and Freezing of Gait in Parkinson’s Disease: Objective Assessment through a Wearable Wireless System. Front. Neurol..

[22] Curtze C., Nutt J.G., Carlson-Kuhta P., Mancini M., Horak F.B. (2015). Levodopa Is a Double-Edged Sword for Balance and Gait in People with Parkinson’s Disease. Mov. Disord..

[23] Vila M.H., Pérez R., Mollinedo I., Cancela J.M. (2021). Analysis of Gait for Disease Stage in Patients with Parkinson’s Disease. Int. J. Environ. Res. Public. Health.

[24] Brognara L., Palumbo P., Grimm B., Palmerini L. (2019). Assessing Gait in Parkinson’s Disease Using Wearable Motion Sensors: A Systematic Review. Diseases.

[25] Marano M., Tinkhauser G., Anzini G., Leogrande G., Ricciuti R., Paniccia M., Belli A., Pierleoni P., Di Lazzaro V., Raggiunto S. (2025). Subthalamic Beta Power and Gait in Parkinson’s Disease during Unsupervised Remote Monitoring. Park. Relat. Disord..

[26] von Elm E., Altman D.G., Egger M., Pocock S.J., Gøtzsche P.C., Vandenbroucke J.P. (2007). The Strengthening the Reporting of Observational Studies in Epidemiology (STROBE) Statement: Guidelines for Reporting Observational Studies. Lancet.

[27] Bhidayasiri R., Tarsy D. (2012). Parkinson’s Disease: Hoehn and Yahr Scale. Movement Disorders: A Video Atlas.

[28] Dalrymple-Alford J.C., MacAskill M.R., Nakas C.T., Livingston L., Graham C., Crucian G.P., Melzer T.R., Kirwan J., Keenan R., Wells S. (2010). The MoCA. Neurology.

[29] Monaghan A.S., Ragothaman A., Harker G.R., Carlson-Kuhta P., Horak F.B., Peterson D.S. (2023). Freezing of Gait in Parkinson’s Disease: Implications for Dual-Task Walking. J. Park. Dis..

[30] Bezdicek O., Růžička F., Fendrych Mazancova A., Roth J., Dušek P., Mueller K., Růžička E., Jech R. (2017). Frontal Assessment Battery in Parkinson’s Disease: Validity and Morphological Correlates. J. Int. Neuropsychol. Soc..

[31] Lucas McKay J., Goldstein F.C., Sommerfeld B., Bernhard D., Perez Parra S., Factor S.A. (2019). Freezing of Gait Can Persist after an Acute Levodopa Challenge in Parkinson’s Disease. npj Park. Dis..

[32] Fabbri M., Coelho M., Abreu D., Guedes L.C., Rosa M.M., Costa N., Antonini A., Ferreira J.J. (2016). Do Patients with Late-Stage Parkinson’s Disease Still Respond to Levodopa?. Park. Relat. Disord..

[33] Saranza G., Lang A.E. (2021). Levodopa Challenge Test: Indications, Protocol, and Guide. J. Neurol..

[34] Tomlinson C.L., Stowe R., Patel S., Rick C., Gray R., Clarke C.E. (2010). Systematic Review of Levodopa Dose Equivalency Reporting in Parkinson’s Disease. Mov. Disord..

[35] Saggio G., Tombolini F., Ruggiero A. (2021). Technology-Based Complex Motor Tasks Assessment: A 6-DOF Inertial-Based System Versus a Gold-Standard Optoelectronic-Based One. IEEE Sens. J..

[36] Ricci M., Terribili M., Giannini F., Errico V., Pallotti A., Galasso C., Tomasello L., Sias S., Saggio G. (2019). Wearable-Based Electronics to Objectively Support Diagnosis of Motor Impairments in School-Aged Children. J. Biomech..

[37] Kwak S., Park S.-H. (2019). Normality Test in Clinical Research. J. Rheum. Dis..

[38] Sheldon M.R., Fillyaw M.J., Thompson W.D. (1996). The Use and Interpretation of the Friedman Test in the Analysis of Ordinal-Scale Data in Repeated Measures Designs. Physiother. Res. Int..

[39] Sedgwick P. (2014). Spearman’s Rank Correlation Coefficient. BMJ.

[40] van Loon W. (2017). The Power of the Benjamini-Hochberg Procedure. Master’s Thesis.

[41] Nikolcheva T., Pagano G., Pross N., Simuni T., Marek K., Postuma R.B., Pavese N., Stocchi F., Seppi K., Monnet A. (2025). A Phase 2b, Multicenter, Randomized, Double-Blind, Placebo-Controlled Study to Evaluate the Efficacy and Safety of Intravenous Prasinezumab in Early-Stage Parkinson’s Disease (PADOVA): Rationale, Design, and Baseline Data. Park. Relat. Disord..

[42] Di Lazzaro G., Ricci M., Saggio G., Costantini G., Schirinzi T., Alwardat M., Pietrosanti L., Patera M., Scalise S., Giannini F. (2021). Technology-Based Therapy-Response and Prognostic Biomarkers in a Prospective Study of a de Novo Parkinson’s Disease Cohort. npj Park. Dis..

[43] Pringsheim T., Day G.S., Smith D.B., Rae-Grant A., Licking N., Armstrong M.J., de Bie R.M.A., Roze E., Miyasaki J.M., Hauser R.A. (2021). Dopaminergic Therapy for Motor Symptoms in Early Parkinson Disease Practice Guideline Summary. Neurology.

[44] Stinear C.M., Byblow W.D. (2014). Predicting and Accelerating Motor Recovery after Stroke. Curr. Opin. Neurol..

[45] Martinu K., Nagano-Saito A., Fogel S., Monchi O. (2014). Asymmetrical Effect of Levodopa on the Neural Activity of Motor Regions in PD. PLoS ONE.

[46] Faria M.H., Simieli L., Rietdyk S., Penedo T., Santinelli F.B., Barbieri F.A. (2023). (A)Symmetry during Gait Initiation in People with Parkinson’s Disease: A Motor and Cortical Activity Exploratory Study. Front. Aging Neurosci..

[47] Lambert K.J.M., Singhal A., Leung A.W.S. (2024). The Lateralized Effects of Parkinson’s Disease on Motor Imagery: Evidence from Mental Chronometry. Brain Cogn..

[48] Adams J.L., Dinesh K., Snyder C.W., Xiong M., Tarolli C.G., Sharma S., Dorsey E.R., Sharma G. (2021). A Real-World Study of Wearable Sensors in Parkinson’s Disease. npj Park. Dis..

[49] Monje M.H.G., Sánchez-Ferro Á., Pineda-Pardo J.A., Vela-Desojo L., Alonso-Frech F., Obeso J.A. (2021). Motor Onset Topography and Progression in Parkinson’s Disease: The Upper Limb Is First. Mov. Disord..

[50] Halliday G., Hely M., Reid W., Morris J. (2008). The Progression of Pathology in Longitudinally Followed Patients with Parkinson’s Disease. Acta Neuropathol..

[51] Bryant M.S., Rintala D.H., Hou J.G., Lai E.C., Protas E.J. (2011). Effects of Levodopa on Forward and Backward Gait Patterns in Persons with Parkinson’s Disease. NeuroRehabilitation.

[52] Fasano A., Mazzoni A., Falotico E. (2022). Reaching and Grasping Movements in Parkinson’s Disease: A Review. J. Park. Dis..

[53] Maillet A., Krainik A., Debû B., Troprès I., Lagrange C., Thobois S., Pollak P., Pinto S. (2012). Levodopa Effects on Hand and Speech Movements in Patients with Parkinson’s Disease: A fMRI Study. PLoS ONE.

[54] Syeda H.B., Glover A., Pillai L., Kemp A.S., Spencer H., Lotia M., Larson-Prior L.J., Virmani T. (2022). Amplitude Setting and Dopamine Response of Finger Tapping and Gait Are Related in Parkinson’s Disease. Sci. Rep..

[55] Shinoda Y., Sugiuchi Y., Izawa Y., Hata Y. (2006). Long Descending Motor Tract Axons and Their Control of Neck and Axial Muscles. Prog. Brain Res..

[56] Thijssen E., Makai-Bölöni S., van Brummelen E., den Heijer J., Yavuz Y., Doll R., Groeneveld G.J. (2022). A Placebo-Controlled Study to Assess the Sensitivity of Finger Tapping to Medication Effects in Parkinson’s Disease. Mov. Disord. Clin. Pract..

[57] Li M.H., Mestre T.A., Fox S.H., Taati B. (2018). Vision-Based Assessment of Parkinsonism and Levodopa-Induced Dyskinesia with Pose Estimation. J. Neuroeng. Rehabil..

[58] Hill A., Cantú H., Côté J.N., Nantel J. (2024). Reaching and Stepping Respond Differently to Medication and Cueing in Parkinson’s Disease. Sci. Rep..

[59] Workman C.D., Thrasher T.A. (2019). The Influence of Dopaminergic Medication on Gait Automaticity in Parkinson’s Disease. J. Clin. Neurosci..

[60] Herz D.M., Brown P. (2023). Moving, Fast and Slow: Behavioural Insights into Bradykinesia in Parkinson’s Disease. Brain.

[61] Zanardi A.P.J., da Silva E.S., Costa R.R., Passos-Monteiro E., dos Santos I.O., Kruel L.F.M., Peyré-Tartaruga L.A. (2021). Gait Parameters of Parkinson’s Disease Compared with Healthy Controls: A Systematic Review and Meta-Analysis. Sci. Rep..

[62] Ramdhani R.A., Watts J., Kline M., Fitzpatrick T., Niethammer M., Khojandi A. (2023). Differential Spatiotemporal Gait Effects with Frequency and Dopaminergic Modulation in STN-DBS. Front. Aging Neurosci..

[63] Ali F., Benarroch E. (2022). What Is the Brainstem Control of Locomotion?. Neurology.

[64] Takakusaki K., Chiba R., Nozu T., Okumura T. (2016). Brainstem Control of Locomotion and Muscle Tone with Special Reference to the Role of the Mesopontine Tegmentum and Medullary Reticulospinal Systems. J. Neural Transm..

[65] Turner R.S., Desmurget M. (2010). Basal Ganglia Contributions to Motor Control: A Vigorous Tutor. Curr. Opin. Neurobiol..

[66] Spraker M.B., Yu H., Corcos D.M., Vaillancourt D.E. (2007). Role of Individual Basal Ganglia Nuclei in Force Amplitude Generation. J. Neurophysiol..

[67] Su Z.H., Patel S., Gavine B., Buchanan T., Bogdanovic M., Sarangmat N., Green A.L., Bloem B.R., FitzGerald J.J., Antoniades C.A. (2023). Deep Brain Stimulation and Levodopa Affect Gait Variability in Parkinson Disease Differently. Neuromodulation Technol. Neural Interface.

[68] Pierleoni P., Raggiunto S., Belli A., Paniccia M., Bazgir O., Palma L. (2022). A Single Wearable Sensor for Gait Analysis in Parkinson’s Disease: A Preliminary Study. Appl. Sci..

[69] Sica M., Tedesco S., Crowe C., Kenny L., Moore K., Timmons S., Barton J., O’Flynn B., Komaris D.-S. (2021). Continuous Home Monitoring of Parkinson’s Disease Using Inertial Sensors: A Systematic Review. PLoS ONE.

[70] di Biase L., Di Santo A., Caminiti M.L., De Liso A., Shah S.A., Ricci L., Di Lazzaro V. (2020). Gait Analysis in Parkinson’s Disease: An Overview of the Most Accurate Markers for Diagnosis and Symptoms Monitoring. Sensors.

[71] Fay-Karmon T., Galor N., Heimler B., Zilka A., Bartsch R.P., Plotnik M., Hassin-Baer S. (2024). Home-Based Monitoring of Persons with Advanced Parkinson’s Disease Using Smartwatch-Smartphone Technology. Sci. Rep..

[72] Adams J.L., Kangarloo T., Gong Y., Khachadourian V., Tracey B., Volfson D., Latzman R.D., Cosman J., Edgerton J., Anderson D. (2024). Using a Smartwatch and Smartphone to Assess Early Parkinson’s Disease in the WATCH-PD Study over 12 Months. npj Park. Dis..

[73] Farabolini G., Baldini N., Pagano A., Andrenelli E., Pepa L., Morone G., Ceravolo M.G., Capecci M. (2025). Continuous Movement Monitoring at Home Through Wearable Devices: A Systematic Review. Sensors.

